# Cannabis and Cognitive Deficiency

**DOI:** 10.1192/j.eurpsy.2022.2125

**Published:** 2022-09-01

**Authors:** S. Brahim, M. Henia, A. Haj Mohamed, R. Chetoui, L. Zarrouk

**Affiliations:** 1 University Hospital of Mahdia, Psychiatry, chebba, Tunisia; 2 University Hospital of Mahdia, Tunisia., Psychiatry, mahdia, Tunisia; 3 University Hospital of Mahdia, Psychiatry, Mahdia, Tunisia; 4 hospital Tahar sfar Mahdia, Department Of Psychiatry Mahdia, monastir, Tunisia

**Keywords:** Cannabis, cognitive deficiency

## Abstract

**Introduction:**

Acute and chronic exposure to cannabis have been associated with neurocognitive deficits in executive function, including inhibitory control processes.

**Objectives:**

To research memory deficiency in the young consumers of cannabis in Tunisia.

**Methods:**

this is a transversal descriptive study conducted during two months (January and February 2020). The research involved about 137 participants in the emergency department at the university hospital of Mahdia

**Results:**

In our study population, there was a noticeable male predominance of 71%. Hence, the age structure ranged between 18 years old and 35 years old. Among the latters, 65.9% were single, and 29.7% experienced school failure. In this sample, 23.2% had a psychiatric history. The average age of the first use of cannabis was between 18 and 25 years old in 70% of cases. Besides, a high percentage of association of other substances was found among cannabis users as follows: use of tobacco 74.6%, alcohol 72.5% ecstasy 41.3%, and cocaine 25.4%. The use of cannabis was considered as a means of indulgence for 66.7% of the study population, as an anxiolytic for 26.8%, and as a sedative for 23.9%. Additionally, the effect of cannabis use on working memory deficiency according to the functional impact assessment scale was: no deficiency in 19% of cannabis users, minimal in 34%, mild in 32%, moderate in 9%, fairly severe in 4%, very severe in 1%, and extreme in1% of cases.

**Conclusions:**

The assumption of the effect of cannabis on memory and cognitive deficiency remains controversial and leads us to suggest further in-depth study of this subject.

**Disclosure:**

No significant relationships.

